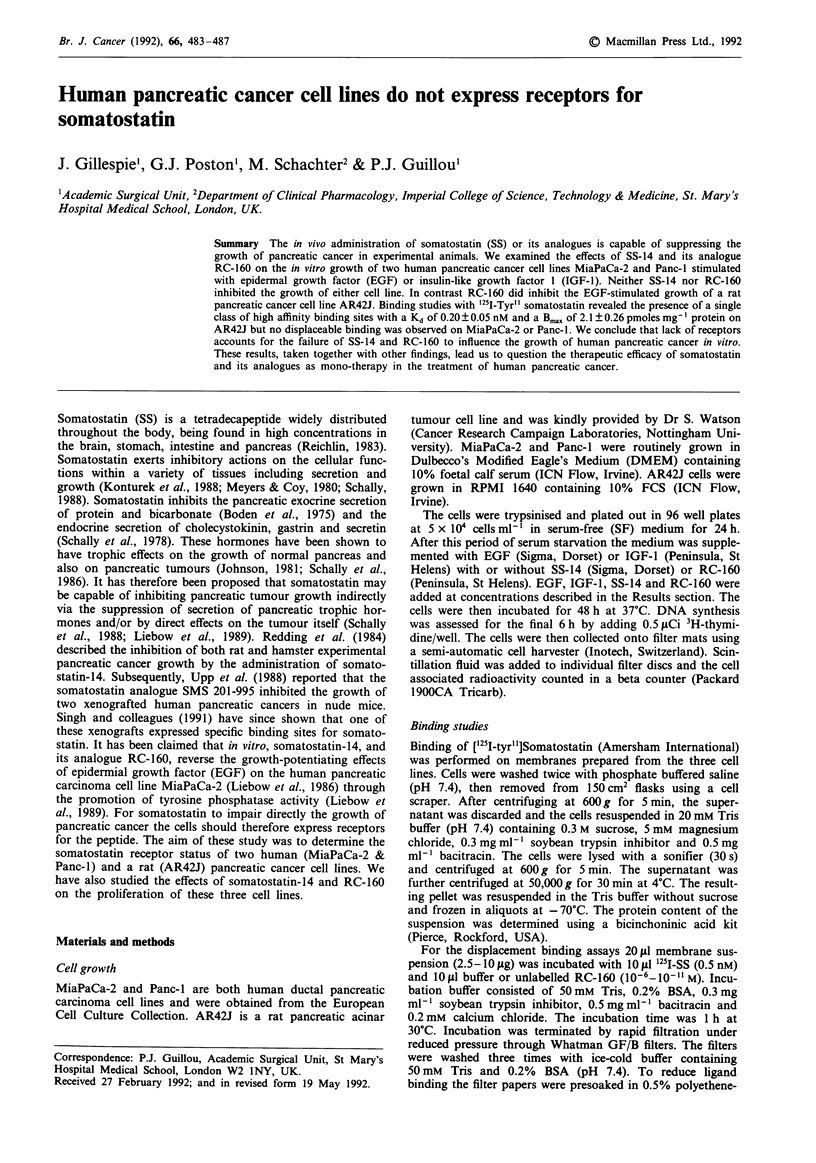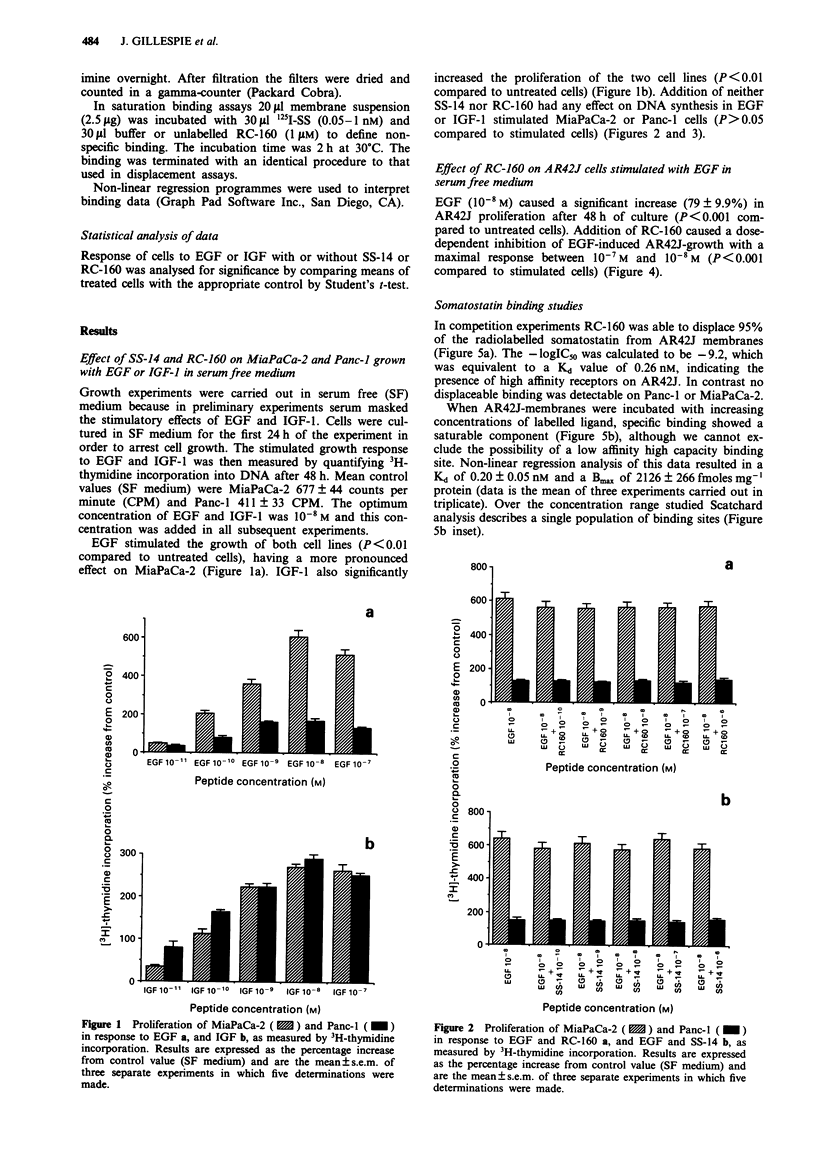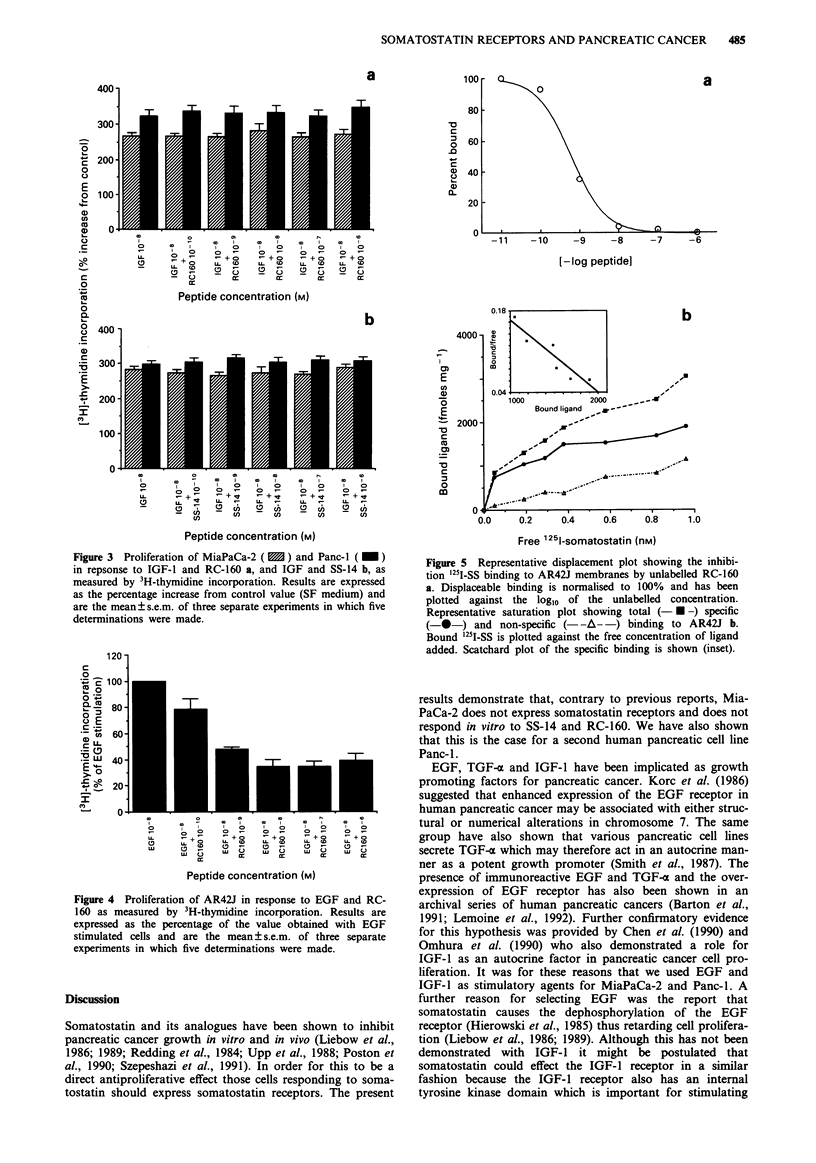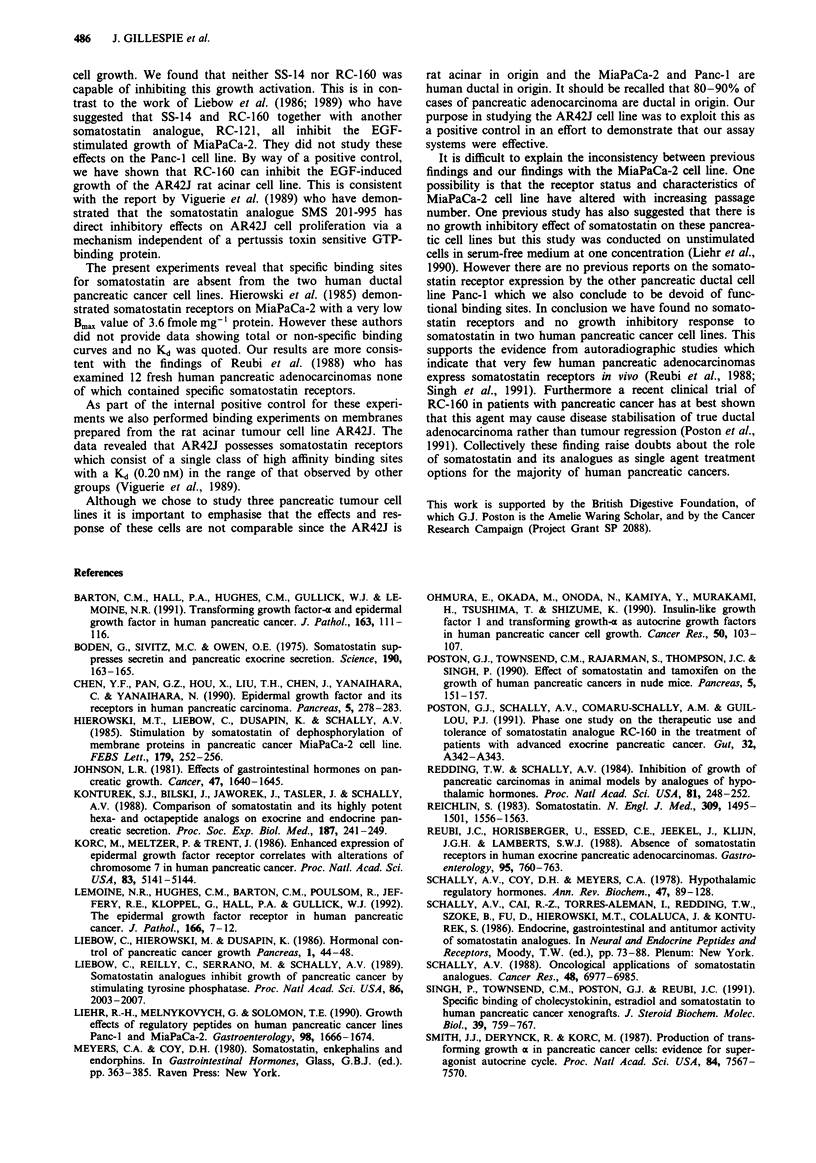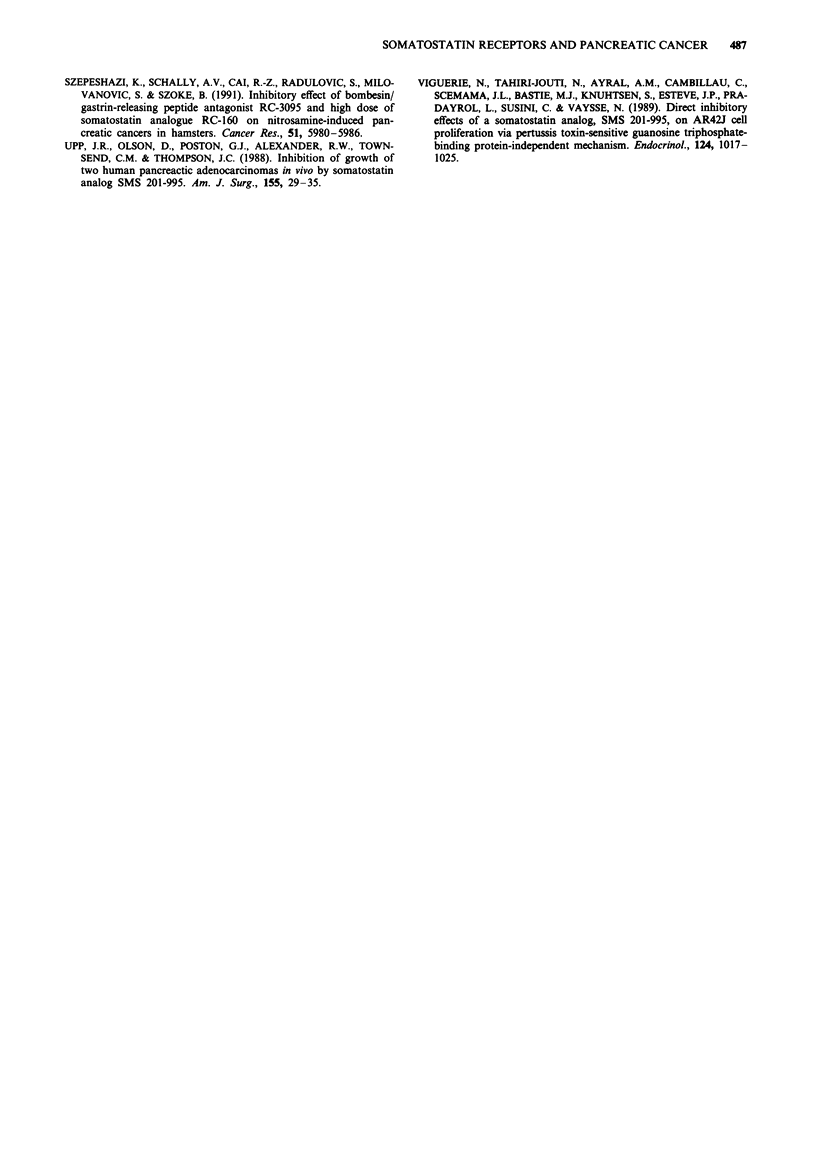# Human pancreatic cancer cell lines do not express receptors for somatostatin.

**DOI:** 10.1038/bjc.1992.300

**Published:** 1992-09

**Authors:** J. Gillespie, G. J. Poston, M. Schachter, P. J. Guillou

**Affiliations:** Academic Surgical Unit, Imperial College of Science, Technology & Medicine, St. Mary's Hospital Medical School, London, UK.

## Abstract

The in vivo administration of somatostatin (SS) or its analogues is capable of suppressing the growth of pancreatic cancer in experimental animals. We examined the effects of SS-14 and its analogue RC-160 on the in vitro growth of two human pancreatic cancer cell lines MiaPaCa-2 and Panc-1 stimulated with epidermal growth factor (EGF) or insulin-like growth factor 1 (IGF-1). Neither SS-14 nor RC-160 inhibited the growth of either cell line. In contrast RC-160 did inhibit the EGF-stimulated growth of a rat pancreatic cancer cell line AR42J. Binding studies with 125I-Tyr11 somatostatin revealed the presence of a single class of high affinity binding sites with a Kd of 0.20 +/- 0.05 nM and a Bmax of 2.1 +/- 0.26 pmoles mg-1 protein on AR42J but not displaceable binding was observed on MiaPaCa-2 or Panc-1. We conclude that lack of receptors accounts for the failure of SS-14 and RC-160 to influence the growth of human pancreatic cancer in vitro. These results, taken together with other findings, lead us to question the therapeutic efficacy of somatostatin and its analogues as mono-therapy in the treatment of human pancreatic cancer.


*Br. J. Cancer* (1992), **66**, 483–487 © Macmillan Press Ltd., 1992

### Human pancreatic cancer cell lines do not express receptors for somatostatin

J. Gillespie[1], G.J. Poston[1], M. Schachter[2] & P.J. Guillou[1]

[1]*Academic Surgical Unit,* [2]*Department of Clinical Pharmacology, Imperial College of Science, Technology & Medicine, St. Mary's Hospital Medical School, London, UK.*

**Summary** The *in vivo* administration of somatostatin (SS) or its analogues is capable of suppressing the growth of pancreatic cancer in experimental animals. We examined the effects of SS-14 and its analogue RC-160 on the *in vitro* growth of two human pancreatic cancer cell lines MiaPaCa-2 and Panc-1 stimulated with epidermal growth factor (EGF) or insulin-like growth factor 1 (IGF-1). Neither SS-14 nor RC-160 inhibited the growth of either cell line. In contrast RC-160 did inhibit the EGF-stimulated growth of a rat pancreatic cancer cell line AR42J. Binding studies with $^{125}$I-Tyr$^{11}$ somatostatin revealed the presence of a single class of high affinity binding sites with a $K_d$ of $0.20 \pm 0.05$ nM and a $B_{max}$ of $2.1 \pm 0.26$ pmoles mg$^{-1}$ protein on AR42J but no displaceable binding was observed on MiaPaCa-2 or Panc-1. We conclude that lack of receptors accounts for the failure of SS-14 and RC-160 to influence the growth of human pancreatic cancer *in vitro*. These results, taken together with other findings, lead us to question the therapeutic efficacy of somatostatin and its analogues as mono-therapy in the treatment of human pancreatic cancer.

Somatostatin (SS) is a tetradecapeptide widely distributed throughout the body, being found in high concentrations in the brain, stomach, intestine and pancreas (Reichlin, 1983). Somatostatin exerts inhibitory actions on the cellular functions within a variety of tissues including secretion and growth (Konturek *et al.*, 1988; Meyers & Coy, 1980; Schally, 1988). Somatostatin inhibits the pancreatic exocrine secretion of protein and bicarbonate (Boden *et al.*, 1975) and the endocrine secretion of cholecystokinin, gastrin and secretin (Schally *et al.*, 1978). These hormones have been shown to have trophic effects on the growth of normal pancreas and also on pancreatic tumours (Johnson, 1981; Schally *et al.*, 1986). It has therefore been proposed that somatostatin may be capable of inhibiting pancreatic tumour growth indirectly via the suppression of secretion of pancreatic trophic hormones and/or by direct effects on the tumour itself (Schally *et al.*, 1988; Liebow *et al.*, 1989). Redding *et al.* (1984) described the inhibition of both rat and hamster experimental pancreatic cancer growth by the administration of somatostatin-14. Subsequently, Upp *et al.* (1988) reported that the somatostatin analogue SMS 201-995 inhibited the growth of two xenografted human pancreatic cancers in nude mice. Singh and colleagues (1991) have since shown that one of these xenografts expressed specific binding sites for somatostatin. It has been claimed that *in vitro*, somatostatin-14, and its analogue RC-160, reverse the growth-potentiating effects of epidermial growth factor (EGF) on the human pancreatic carcinoma cell line MiaPaCa-2 (Liebow *et al.*, 1986) through the promotion of tyrosine phosphatase activity (Liebow *et al.*, 1989). For somatostatin to impair directly the growth of pancreatic cancer the cells should therefore express receptors for the peptide. The aim of these study was to determine the somatostatin receptor status of two human (MiaPaCa-2 & Panc-1) and a rat (AR42J) pancreatic cancer cell lines. We have also studied the effects of somatostatin-14 and RC-160 on the proliferation of these three cell lines.

### Materials and methods

### *Cell growth*

MiaPaCa-2 and Panc-1 are both human ductal pancreatic carcinoma cell lines and were obtained from the European Cell Culture Collection. AR42J is a rat pancreatic acinar tumour cell line and was kindly provided by Dr S. Watson (Cancer Research Campaign Laboratories, Nottingham University). MiaPaCa-2 and Panc-1 were routinely grown in Dulbecco's Modified Eagle's Medium (DMEM) containing 10% foetal calf serum (ICN Flow, Irvine). AR42J cells were grown in RPMI 1640 containing 10% FCS (ICN Flow, Irvine).

The cells were trypsinised and plated out in 96 well plates at $5 \times 10^4$ cells ml$^{-1}$ in serum-free (SF) medium for 24 h. After this period of serum starvation the medium was supplemented with EGF (Sigma, Dorset) or IGF-1 (Peninsula, St Helens) with or without SS-14 (Sigma, Dorset) or RC-160 (Peninsula, St Helens). EGF, IGF-1, SS-14 and RC-160 were added at concentrations described in the Results section. The cells were then incubated for 48 h at 37°C. DNA synthesis was assessed for the final 6 h by adding 0.5 μCi $^3$H-thymidine/well. The cells were then collected onto filter mats using a semi-automatic cell harvester (Inotech, Switzerland). Scintillation fluid was added to individual filter discs and the cell associated radioactivity counted in a beta counter (Packard 1900CA Tricarb).

### *Binding studies*

Binding of [$^{125}$I-tyr$^{11}$]Somatostatin (Amersham International) was performed on membranes prepared from the three cell lines. Cells were washed twice with phosphate buffered saline (pH 7.4), then removed from 150 cm$^2$ flasks using a cell scraper. After centrifuging at 600 *g* for 5 min, the supernatant was discarded and the cells resuspended in 20 mM Tris buffer (pH 7.4) containing 0.3 M sucrose, 5 mM magnesium chloride, 0.3 mg ml$^{-1}$ soybean trypsin inhibitor and 0.5 mg ml$^{-1}$ bacitracin. The cells were lysed with a sonifier (30 s) and centrifuged at 600 *g* for 5 min. The supernatant was further centrifuged at 50,000 *g* for 30 min at 4°C. The resulting pellet was resuspended in the Tris buffer without sucrose and frozen in aliquots at −70°C. The protein content of the suspension was determined using a bicinchoninic acid kit (Pierce, Rockford, USA).

For the displacement binding assays 20 μl membrane suspension (2.5–10 μg) was incubated with 10 μl $^{125}$I-SS (0.5 nM) and 10 μl buffer or unlabelled RC-160 ($10^{-6}$–$10^{-11}$ M). Incubation buffer consisted of 50 mM Tris, 0.2% BSA, 0.3 mg ml$^{-1}$ soybean trypsin inhibitor, 0.5 mg ml$^{-1}$ bacitracin and 0.2 mM calcium chloride. The incubation time was 1 h at 30°C. Incubation was terminated by rapid filtration under reduced pressure through Whatman GF/B filters. The filters were washed three times with ice-cold buffer containing 50 mM Tris and 0.2% BSA (pH 7.4). To reduce ligand binding the filter papers were presoaked in 0.5% polyethene-

Correspondence: P.J. Guillou, Academic Surgical Unit, St Mary's Hospital Medical School, London W2 1NY, UK.
Received 27 February 1992; and in revised form 19 May 1992.

imine overnight. After filtration the filters were dried and counted in a gamma-counter (Packard Cobra).

In saturation binding assays 20 µl membrane suspension (2.5 µg) was incubated with 30 µl $^{125}$I-SS (0.05–1 nM) and 30 µl buffer or unlabelled RC-160 (1 µM) to define non-specific binding. The incubation time was 2 h at 30°C. The binding was terminated with an identical procedure to that used in displacement assays.

Non-linear regression programmes were used to interpret binding data (Graph Pad Software Inc., San Diego, CA).

*Statistical analysis of data*

Response of cells to EGF or IGF with or without SS-14 or RC-160 was analysed for significance by comparing means of treated cells with the appropriate control by Student's *t*-test.

### Results

*Effect of SS-14 and RC-160 on MiaPaCa-2 and Panc-1 grown with EGF or IGF-1 in serum free medium*

Growth experiments were carried out in serum free (SF) medium because in preliminary experiments serum masked the stimulatory effects of EGF and IGF-1. Cells were cultured in SF medium for the first 24 h of the experiment in order to arrest cell growth. The stimulated growth response to EGF and IGF-1 was then measured by quantifying $^3$H-thymidine incorporation into DNA after 48 h. Mean control values (SF medium) were MiaPaCa-2 677 ± 44 counts per minute (CPM) and Panc-1 411 ± 33 CPM. The optimum concentration of EGF and IGF-1 was $10^{-8}$ M and this concentration was added in all subsequent experiments.

EGF stimulated the growth of both cell lines ($P<0.01$ compared to untreated cells), having a more pronounced effect on MiaPaCa-2 (Figure 1a). IGF-1 also significantly increased the proliferation of the two cell lines ($P<0.01$ compared to untreated cells) (Figure 1b). Addition of neither SS-14 nor RC-160 had any effect on DNA synthesis in EGF or IGF-1 stimulated MiaPaCa-2 or Panc-1 cells ($P>0.05$ compared to stimulated cells) (Figures 2 and 3).

*Effect of RC-160 on AR42J cells stimulated with EGF in serum free medium*

EGF ($10^{-8}$ M) caused a significant increase (79 ± 9.9%) in AR42J proliferation after 48 h of culture ($P<0.001$ compared to untreated cells). Addition of RC-160 caused a dose-dependent inhibition of EGF-induced AR42J-growth with a maximal response between $10^{-7}$ M and $10^{-8}$ M ($P<0.001$ compared to stimulated cells) (Figure 4).

*Somatostatin binding studies*

In competition experiments RC-160 was able to displace 95% of the radiolabelled somatostatin from AR42J membranes (Figure 5a). The $-\log IC_{50}$ was calculated to be −9.2, which was equivalent to a $K_d$ value of 0.26 nM, indicating the presence of high affinity receptors on AR42J. In contrast no displaceable binding was detectable on Panc-1 or MiaPaCa-2.

When AR42J-membranes were incubated with increasing concentrations of labelled ligand, specific binding showed a saturable component (Figure 5b), although we cannot exclude the possibility of a low affinity high capacity binding site. Non-linear regression analysis of this data resulted in a $K_d$ of 0.20 ± 0.05 nM and a $B_{max}$ of 2126 ± 266 fmoles mg$^{-1}$ protein (data is the mean of three experiments carried out in triplicate). Over the concentration range studied Scatchard analysis describes a single population of binding sites (Figure 5b inset).

**Figure 1** Proliferation of MiaPaCa-2 ( ▨ ) and Panc-1 ( ■ ) in response to EGF **a**, and IGF **b**, as measured by $^3$H-thymidine incorporation. Results are expressed as the percentage increase from control value (SF medium) and are the mean ± s.e.m. of three separate experiments in which five determinations were made.

**Figure 2** Proliferation of MiaPaCa-2 ( ▨ ) and Panc-1 ( ■ ) in response to EGF and RC-160 **a**, and EGF and SS-14 **b**, as measured by $^3$H-thymidine incorporation. Results are expressed as the percentage increase from control value (SF medium) and are the mean ± s.e.m. of three separate experiments in which five determinations were made.

**Figure 3** Proliferation of MiaPaCa-2 ( ▨ ) and Panc-1 ( ■ ) in repsonse to IGF-1 and RC-160 **a**, and IGF and SS-14 **b**, as measured by $^{3}$H-thymidine incorporation. Results are expressed as the percentage increase from control value (SF medium) and are the mean ± s.e.m. of three separate experiments in which five determinations were made.

**Figure 4** Proliferation of AR42J in response to EGF and RC-160 as measured by $^{3}$H-thymidine incorporation. Results are expressed as the percentage of the value obtained with EGF stimulated cells and are the mean ± s.e.m. of three separate experiments in which five determinations were made.

**Figure 5** Representative displacement plot showing the inhibition $^{125}$I-SS binding to AR42J membranes by unlabelled RC-160 **a**. Displaceable binding is normalised to 100% and has been plotted against the $\log_{10}$ of the unlabelled concentration. Representative saturation plot showing total (— ■ -) specific (—●—) and non-specific (— –Δ– —) binding to AR42J **b**. Bound $^{125}$I-SS is plotted against the free concentration of ligand added. Scatchard plot of the specific binding is shown (inset).

### Discussion

Somatostatin and its analogues have been shown to inhibit pancreatic cancer growth *in vitro* and *in vivo* (Liebow *et al.*, 1986; 1989; Redding *et al.*, 1984; Upp *et al.*, 1988; Poston *et al.*, 1990; Szepeshazi *et al.*, 1991). In order for this to be a direct antiproliferative effect those cells responding to somatostatin should express somatostatin receptors. The present results demonstrate that, contrary to previous reports, MiaPaCa-2 does not express somatostatin receptors and does not respond *in vitro* to SS-14 and RC-160. We have also shown that this is the case for a second human pancreatic cell line Panc-1.

EGF, TGF-α and IGF-1 have been implicated as growth promoting factors for pancreatic cancer. Korc *et al.* (1986) suggested that enhanced expression of the EGF receptor in human pancreatic cancer may be associated with either structural or numerical alterations in chromosome 7. The same group have also shown that various pancreatic cell lines secrete TGF-α which may therefore act in an autocrine manner as a potent growth promoter (Smith *et al.*, 1987). The presence of immunoreactive EGF and TGF-α and the overexpression of EGF receptor has also been shown in an archival series of human pancreatic cancers (Barton *et al.*, 1991; Lemoine *et al.*, 1992). Further confirmatory evidence for this hypothesis was provided by Chen *et al.* (1990) and Omhura *et al.* (1990) who also demonstrated a role for IGF-1 as an autocrine factor in pancreatic cancer cell proliferation. It was for these reasons that we used EGF and IGF-1 as stimulatory agents for MiaPaCa-2 and Panc-1. A further reason for selecting EGF was the report that somatostatin causes the dephosphorylation of the EGF receptor (Hierowski *et al.*, 1985) thus retarding cell proliferation (Liebow *et al.*, 1986; 1989). Although this has not been demonstrated with IGF-1 it might be postulated that somatostatin could effect the IGF-1 receptor in a similar fashion because the IGF-1 receptor also has an internal tyrosine kinase domain which is important for stimulating cell growth. We found that neither SS-14 nor RC-160 was capable of inhibiting this growth activation. This is in contrast to the work of Liebow *et al.* (1986; 1989) who have suggested that SS-14 and RC-160 together with another somatostatin analogue, RC-121, all inhibit the EGF-stimulated growth of MiaPaCa-2. They did not study these effects on the Panc-1 cell line. By way of a positive control, we have shown that RC-160 can inhibit the EGF-induced growth of the AR42J rat acinar cell line. This is consistent with the report by Viguerie *et al.* (1989) who have demonstrated that the somatostatin analogue SMS 201-995 has direct inhibitory effects on AR42J cell proliferation via a mechanism independent of a pertussis toxin sensitive GTP-binding protein.

The present experiments reveal that specific binding sites for somatostatin are absent from the two human ductal pancreatic cancer cell lines. Hierowski *et al.* (1985) demonstrated somatostatin receptors on MiaPaCa-2 with a very low $B_{max}$ value of 3.6 fmole $mg^{-1}$ protein. However these authors did not provide data showing total or non-specific binding curves and no $K_d$ was quoted. Our results are more consistent with the findings of Reubi *et al.* (1988) who has examined 12 fresh human pancreatic adenocarcinomas none of which contained specific somatostatin receptors.

As part of the internal positive control for these experiments we also performed binding experiments on membranes prepared from the rat acinar tumour cell line AR42J. The data revealed that AR42J possesses somatostatin receptors which consist of a single class of high affinity binding sites with a $K_d$ (0.20 nM) in the range of that observed by other groups (Viguerie *et al.*, 1989).

Although we chose to study three pancreatic tumour cell lines it is important to emphasise that the effects and response of these cells are not comparable since the AR42J is rat acinar in origin and the MiaPaCa-2 and Panc-1 are human ductal in origin. It should be recalled that 80–90% of cases of pancreatic adenocarcinoma are ductal in origin. Our purpose in studying the AR42J cell line was to exploit this as a positive control in an effort to demonstrate that our assay systems were effective.

It is difficult to explain the inconsistency between previous findings and our findings with the MiaPaCa-2 cell line. One possibility is that the receptor status and characteristics of MiaPaCa-2 cell line have altered with increasing passage number. One previous study has also suggested that there is no growth inhibitory effect of somatostatin on these pancreatic cell lines but this study was conducted on unstimulated cells in serum-free medium at one concentration (Liehr *et al.*, 1990). However there are no previous reports on the somatostatin receptor expression by the other pancreatic ductal cell line Panc-1 which we also conclude to be devoid of functional binding sites. In conclusion we have found no somatostatin receptors and no growth inhibitory response to somatostatin in two human pancreatic cancer cell lines. This supports the evidence from autoradiographic studies which indicate that very few human pancreatic adenocarcinomas express somatostatin receptors *in vivo* (Reubi *et al.*, 1988; Singh *et al.*, 1991). Furthermore a recent clinical trial of RC-160 in patients with pancreatic cancer has at best shown that this agent may cause disease stabilisation of true ductal adenocarcinoma rather than tumour regression (Poston *et al.*, 1991). Collectively these finding raise doubts about the role of somatostatin and its analogues as single agent treatment options for the majority of human pancreatic cancers.

This work is supported by the British Digestive Foundation, of which G.J. Poston is the Amelie Waring Scholar, and by the Cancer Research Campaign (Project Grant SP 2088).

### References

BARTON, C.M., HALL, P.A., HUGHES, C.M., GULLICK, W.J. & LEMOINE, N.R. (1991). Transforming growth factor-α and epidermal growth factor in human pancreatic cancer. *J. Pathol.*, **163**, 111–116.

BODEN, G., SIVITZ, M.C. & OWEN, O.E. (1975). Somatostatin suppresses secretin and pancreatic exocrine secretion. *Science*, **190**, 163–165.

CHEN, Y.F., PAN, G.Z., HOU, X., LIU, T.H., CHEN, J., YANAIHARA, C. & YANAIHARA, N. (1990). Epidermal growth factor and its receptors in human pancreatic carcinoma. *Pancreas*, **5**, 278–283.

HIEROWSKI, M.T., LIEBOW, C., DUSAPIN, K. & SCHALLY, A.V. (1985). Stimulation by somatostatin of dephosphorylation of membrane proteins in pancreatic cancer MiaPaCa-2 cell line. *FEBS Lett.*, **179**, 252–256.

JOHNSON, L.R. (1981). Effects of gastrointestinal hormones on pancreatic growth. *Cancer*, **47**, 1640–1645.

KONTUREK, S.J., BILSKI, J., JAWOREK, J., TASLER, J. & SCHALLY, A.V. (1988). Comparison of somatostatin and its highly potent hexa- and octapeptide analogs on exocrine and endocrine pancreatic secretion. *Proc. Soc. Exp. Biol. Med.*, **187**, 241–249.

KORC, M., MELTZER, P. & TRENT, J. (1986). Enhanced expression of epidermal growth factor receptor correlates with alterations of chromosome 7 in human pancreatic cancer. *Proc. Natl. Acad. Sci. USA*, **83**, 5141–5144.

LEMOINE, N.R., HUGHES, C.M., BARTON, C.M., POULSOM, R., JEFFERY, R.E., KLOPPEL, G., HALL, P.A. & GULLICK, W.J. (1992). The epidermal growth factor receptor in human pancreatic cancer. *J. Pathol.*, **166**, 7–12.

LIEBOW, C., HIEROWSKI, M. & DUSAPIN, K. (1986). Hormonal control of pancreatic cancer growth *Pancreas*, **1**, 44–48.

LIEBOW, C., REILLY, C., SERRANO, M. & SCHALLY, A.V. (1989). Somatostatin analogues inhibit growth of pancreatic cancer by stimulating tyrosine phosphatase. *Proc. Natl Acad. Sci. USA*, **86**, 2003–2007.

LIEHR, R.-H., MELNYKOVYCH, G. & SOLOMON, T.E. (1990). Growth effects of regulatory peptides on human pancreatic cancer lines Panc-1 and MiaPaCa-2. *Gastroenterology*, **98**, 1666–1674.

MEYERS, C.A. & COY, D.H. (1980). Somatostatin, enkephalins and endorphins. In *Gastrointestinal Hormones*, Glass, G.B.J. (ed.). pp. 363–385. Raven Press: New York.

OHMURA, E., OKADA, M., ONODA, N., KAMIYA, Y., MURAKAMI, H., TSUSHIMA, T. & SHIZUME, K. (1990). Insulin-like growth factor 1 and transforming growth-α as autocrine growth factors in human pancreatic cancer cell growth. *Cancer Res.*, **50**, 103–107.

POSTON, G.J., TOWNSEND, C.M., RAJARMAN, S., THOMPSON, J.C. & SINGH, P. (1990). Effect of somatostatin and tamoxifen on the growth of human pancreatic cancers in nude mice. *Pancreas*, **5**, 151–157.

POSTON, G.J., SCHALLY, A.V., COMARU-SCHALLY, A.M. & GUILLOU, P.J. (1991). Phase one study on the therapeutic use and tolerance of somatostatin analogue RC-160 in the treatment of patients with advanced exocrine pancreatic cancer. *Gut*, **32**, A342–A343.

REDDING, T.W. & SCHALLY, A.V. (1984). Inhibition of growth of pancreatic carcinomas in animal models by analogues of hypothalamic hormones. *Proc. Natl Acad. Sci. USA*, **81**, 248–252.

REICHLIN, S. (1983). Somatostatin. *N. Engl. J. Med.*, **309**, 1495–1501, 1556–1563.

REUBI, J.C., HORISBERGER, U., ESSED, C.E., JEEKEL, J., KLIJN, J.G.H. & LAMBERTS, S.W.J. (1988). Absence of somatostatin receptors in human exocrine pancreatic adenocarcinomas. *Gastroenterology*, **95**, 760–763.

SCHALLY, A.V., COY, D.H. & MEYERS, C.A. (1978). Hypothalamic regulatory hormones. *Ann. Rev. Biochem.*, **47**, 89–128.

SCHALLY, A.V., CAI, R.-Z., TORRES-ALEMAN, I., REDDING, T.W., SZOKE, B., FU, D., HIEROWSKI, M.T., COLALUCA, J. & KONTUREK, S. (1986). Endocrine, gastrointestinal and antitumor activity of somatostatin analogues. In *Neural and Endocrine Peptides and Receptors*, Moody, T.W. (ed.), pp. 73–88. Plenum: New York.

SCHALLY, A.V. (1988). Oncological applications of somatostatin analogues. *Cancer Res.*, **48**, 6977–6985.

SINGH, P., TOWNSEND, C.M., POSTON, G.J. & REUBI, J.C. (1991). Specific binding of cholecystokinin, estradiol and somatostatin to human pancreatic cancer xenografts. *J. Steroid Biochem. Molec. Biol.*, **39**, 759–767.

SMITH, J.J., DERYNCK, R. & KORC, M. (1987). Production of transforming growth α in pancreatic cancer cells: evidence for superagonist autocrine cycle. *Proc. Natl Acad. Sci. USA*, **84**, 7567–7570.

SZEPESHAZI, K., SCHALLY, A.V., CAI, R.-Z., RADULOVIC, S., MILOVANOVIC, S. & SZOKE, B. (1991). Inhibitory effect of bombesin/gastrin-releasing peptide antagonist RC-3095 and high dose of somatostatin analogue RC-160 on nitrosamine-induced pancreatic cancers in hamsters. *Cancer Res.*, **51**, 5980–5986.

UPP, J.R., OLSON, D., POSTON, G.J., ALEXANDER, R.W., TOWNSEND, C.M. & THOMPSON, J.C. (1988). Inhibition of growth of two human pancreactic adenocarcinomas *in vivo* by somatostatin analog SMS 201-995. *Am. J. Surg.*, **155**, 29–35.

VIGUERIE, N., TAHIRI-JOUTI, N., AYRAL, A.M., CAMBILLAU, C., SCEMAMA, J.L., BASTIE, M.J., KNUHTSEN, S., ESTEVE, J.P., PRADAYROL, L., SUSINI, C. & VAYSSE, N. (1989). Direct inhibitory effects of a somatostatin analog, SMS 201-995, on AR42J cell proliferation via pertussis toxin-sensitive guanosine triphosphate-binding protein-independent mechanism. *Endocrinol.*, **124**, 1017–1025.